# New variant identified in major susceptibility locus to tuberculosis on chromosomal region 8q12-q13 in Moroccan population: a case control study

**DOI:** 10.1186/s12879-017-2807-9

**Published:** 2017-11-07

**Authors:** Mounia Qrafli, Imane Asekkaj, Jamal Eddine Bourkadi, Rajae El Aouad, Khalid Sadki

**Affiliations:** 1 Physiopathology Team, Immunogenetics and Bioinformatics Unit, Genomic Center of Human Pathologies, Faculty of Sciences, Mohammed V University, Rabat, Morocco; 2 Académie Hassan II des Sciences et Techniques, Rabat, Morocco; 3 Pneumo-Phtisiology Department, Moulay Youssef Hospital, CHU of Rabat, Rabat, Morocco

**Keywords:** Tuberculosis, *NSMAF*, Moroccan, Apoptosis

## Abstract

**Background:**

Tuberculosis (TB) remains a global health problem. Several studies have implicated genetic host factors in predisposing populations to TB disease. In this study, we have selected *NSMAF* (Neutral Sphingomyelinase Activation Associated Factor) as a candidate gene to evaluate its level of association with TB disease in a Moroccan population for two reasons: first, this gene is located in a major susceptibility locus on chromosomal region 8q12-q13 in the Moroccan population, closely linked to the *CYP7A1* gene, which was previously shown to be associated with TB disease; second, *NSMAF* has an important role in immune system function.

**Methods:**

We conducted a case-control study including 269 genomic DNA samples extracted from pulmonary TB (PTB) patients and healthy controls (HC). We genotyped three selected SNPs (rs2228505, rs36067275 and rs10505004) using TaqMan® allelic discrimination assays.

**Results:**

Only the rs1050504 C > T genotype was observed to be significantly associated with an increased risk for developing pulmonary TB (41.8% vs 27%, OR 1.95, 95% CI 1.16–3.27; *p* = 0.01). In contrast, the TT genotype was significantly associated with resistance to PTB (4.1% vs 15.6%, OR 0.23, 95% CI 0.08–0.63; *p* = 0.002).

**Conclusion:**

Our findings suggest that genetic variations in the *NSMAF* gene could modulate the risk of PTB development in a Moroccan population. Further functional studies are needed to confirm these findings.

## Background

Tuberculosis (TB) is one of the oldest infectious diseases that is still a serious health challenge in the developing world. According to a recent report published by the World Health Organization, TB killed 1.5 million people in 2015 [1]. In Morocco, the Ministry of Health registered high incidence in 2015, reaching 89 new cases per 100,000 inhabitants [2].

TB is a multifactorial disease, and thus, identifying host genes that determine its susceptibility is far from an easy task. For this reason, candidate gene studies are receiving increasing attention in genetic epidemiology. This method begins with the selection of a putative candidate gene followed by the selection of genetic polymorphisms based on its predicted function. Hence, by focusing directly on genetic variations within a gene of interest, this approach offers considerable advantages in terms of detecting disease-associated genes [3–5].

In the Moroccan population, a few studies have been conducted in this context, with some reporting a significant association between TB disease and genetic variants in *MIF*, *PTPN22*, *VDR*, *CYP7A1* and *STAT4* [6–10]. Furthermore, it seems that the major susceptibility locus for TB in the Moroccan population is located on chromosomal region 8q12-q13 [11].

**Fig. 1:**
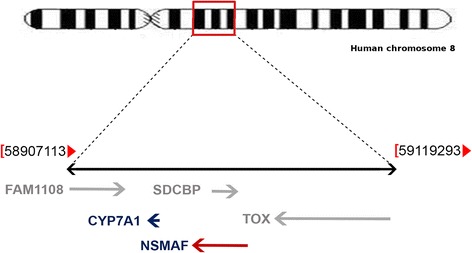
A representative scheme showing the localization of the *NSMAF* gene in the chromosome 8 (modified from [13]). At the chromosomal region 8q12.13 from 58,907,113 to 59,119,293 bp, *NSMAF* gene is closely located between *CYP7A1* and *TOX* gene

Two genetic variants associated with TB have been identified. The first one is rs3808607 in the *CYP7A1* gene, and the second is rs1568952, located 6 kb downstream of the last *TOX* gene exon. The *NSMAF* gene is located [12] between the *CYP7A1* and *TOX* genes (Fig. 1).

The *NSMAF* (Neutral Sphingomyelinase Activation Associated Factor) gene encodes for the protein NSMAF or FAN (factor associated with neutral sphingomyelinase activation) [14]. NSMAF is a 140 KDa WD-repeat protein composed of 917 aa [15, 16]. This protein interacts specifically with the cytoplasmic sphingomyelinase activation domain of the 55kD tumour necrosis factor receptor, known as Tumour Necrosis Factor Receptor Type I (*TNFRI*) or p55(CD120a), also called NSD (neutral sphingomyelinase domain). Indeed, the FAN protein is an essential component of the activation of TNF-α induced neutral sphingomyelinase 2 and 3 [17, 18], leading to activation of the sphingomyelin-ceramide pathway. In fact, this biological event is initiated by the hydrolysis of sphingomyelin to ceramide, the lipid second messenger implicated in cell signalling [19].

In addition, with stable expression of a dominant-negative form of FAN in human fibroblasts, caspase activation and cytochrome c release from mitochondria are reduced, and the TNF-α-triggered apoptosis is obviously inhibited [20]. Furthermore, FAN contributes to the inflammation process and is required for full expression of the genes encoding CXCL2 and IL-6 [21]. Overexpression of FAN in rat cardiomyocytes was shown to lead to increased cell death [22]. Interestingly, *NSMAF* is also associated with multiple sclerosis [23, 24], and one study demonstrated that FAN can promote melanoma cellular motility and tumour invasiveness in an in vivo model [25].

Taking all these findings together, there are several lines of evidence to support the possible involvement of *NSMAF* in TB development; therefore, it could be postulated as a candidate gene of susceptibility in TB disease. The aim of this work is to evaluate the impact of selected *NSMAF* polymorphisms on TB susceptibility in the Moroccan population.

## Methods

### Study design

We conducted a case–control study in the Moroccan population. 10 ml of peripheral blood was collected in ethylenediaminetetraacetic acid tubes from all participants recruited over the course of 2 years. The blood samples were collected from 19 health centres comprising the Centres of TB Treatment and Respiratory Disease (CTRD) and the university hospitals for PTB patients. Patients were evaluated by microbiological diagnosis, physical examination and chest X-rays; all patients were smear positive for acid-fast bacilli and mycobacterial culture and were tuberculin skin test positive. HC group consisted of healthy donors with no signs, symptoms or history of previous tuberculosis. They were tuberculin skin negative and remained in this immunological status during the 2 years after recruitment in a posterior telephonic “check-contact”. The recruitment was done from the Regional Centers of Blood Transfusion (RCBT) of five different regions of Morocco (Oujda, Fez, Tangier, Rabat, and Marrakech). All subjects were negatives for HIV-1/2 infection (tested by Axsym Assays, Abbott Laboratories, Chicago, IL, USA). This study was approved by the local ethics committee (faculty of Medicine and Pharmacy, Mohammed V University of Rabat, as reference number 1169), and informed written consent was obtained from all subjects. Structured questionnaires were used to collect medical history data, biological investigations and demographic parameters (Table 1 **)**. The questionnaire analyses show that the sex ratio (males /females) among TB patients was almost similar to that of the HC group (2.76 vs 2.52, respectively). The mean age ± standard deviation was 33.43 (±13.24) years, ranging from 18 to 67 years, and 32.41 (±11.10) years, ranging from 18 to 61 years, for patients and HC, respectively (Table 1).

**Table 1:** Demographic and clinical characteristics of tuberculosis patients and healthy controls

Variables	TB patients *N* = 128		Healthy controls *N* = 141	
Age (Mean age ± SD)	33,43 ± 13.24		32.41 ± 11.10	
Gender [n (%)]				
Men	94	73.4%	101	71.6%
Women	34	26.6%	40	28.4%
Clinical feature				
Hemoptysis	55/128	43%		
Expectoration	117/128	91%		
Fever	112/128	87%		
Cough	125/128	98%		
weight loss	122/128	95%		
Abnormality shown by chest X-ray examination	128/128	100%		

### *NSMAF* genotyping

DNA extraction: Total genomic DNA was extracted from the peripheral blood of all TB patients and HC using a QIAampDNA Blood Maxi kit (QIAamp**®** DNA Blood Mini Kit, Qiagen GmbH, Hilden, Germany) and was stored at −20 °C until use.

*NSMAF* selected SNPs: Three SNPs of the *NSMAF* gene were selected for genotyping. The first SNP, rs36067275 C > T, is a missense mutation causing a substitution of glutamic acid by lysine at 487. The second SNP, rs2228505 T > C, is a missense mutation causing a substitution of tyrosine by cysteine at position 626. The third is located in the 3′ UTR (untranslated region) represented by rs1050504 C > T.

Real time PCR genotyping: The three selected SNPs were genotyped by Real time PCR technology using a TaqMan® Genotyping Assay (Applied Biosystems, Foster City, CA). Reactions were performed as recommended by the manufacturer (Applied Biosystems). PCR allelic discrimination was performed on a 7500 Fast Real-Time PCR System (Applied Biosystems, Foster City, CA, USA), which measured the specific allele fluorescence of each sample. Impaired samples were genotyped twice.

#### Statistical analysis

Allele and genotype frequencies between PTB patient and HC were determined by direct counting and were compared afterward using the χ2-test. Patients and controls were tested for conformity to Hardy–Weinberg equilibrium. All statistical analyses were performed using EPI INFOTM, version 7.1.0.6 (Centers for Disease Control and Prevention, Atlanta, GA; 08 September 2012). We considered the results with corresponding *p*-values below 0.05 to be statistically significant. Moreover, the odds ratio (OR) with a 95% confidence interval (CI) was calculated to evaluate the risk of association between genotypes or alleles and TB disease.

To estimate the haplotype frequencies, the program CubEX was used [26]. This program is also able to provide the normalized linkage disequilibrium (LD) parameter (D’) and the LD correlation coefficient between two loci (r2).

## Results

All genotype frequencies of patients and HC were consistent with respect to Hardy-Weinberg equilibrium.

There were no significant differences between patients and HC in the distribution of genotypes and allele frequencies for either rs36067275 C/T or rs2228505 T/C SNPs (Table 2). Nevertheless, despite the significant difference not being reached for allele distribution between the groups for rs2228505 T/C SNP, the mutant allele C was more frequent in patients than in controls (14.06% in patients versus 9.32% in controls, *p* = 0.1, OR = 1.59, 95% CI = 0.9–2.79) (Table 3). However, a statistical analysis of genotype distribution for the rs1050504 polymorphism yielded an interesting result. A significant and positive association was found between the CT genotype and an increased risk of PTB development (41.8% vs 27%, OR 1.95, 95% CI 1.16–3.27; *p* = 0.01). Conversely, the TT genotype frequency was statistically more frequent in HC than in patients (15.6% vs 4.1%, OR 0.23, 95% CI 0.08–0.63; *p* = 0.002, respectively) (Table 4).

**Table 2:** Genotypes and alleles frequencies for the rs36067275 polymorphism in pulmonary tuberculosis patients and healthy controls

Patients vs Controls				
rs36067275 [C/T]	PTB *N* = 112 (%)	HC *N* = 116 (%)	*p*-value	OR (95% CI)
Genotypes (N)				
*CC*	112(100)	116(100)	> 0,05	
*CT*	0	0		
*TT*	0	0		
Alleles (2 N)				
*C*	224 (100)	232 (100)	> 0,05	
*T*	0	0		

*CI* confidence interval, *OR* odds ratio

**Table 3:** Genotypes and alleles frequencies for the rs2228505 polymorphism in pulmonary tuberculosis patients and healthy controls

Patients vs Controls				
rs2228505 [T/C]	PTB N = 128 (%)	HC *N* = 118 (%)	*p*-value	OR (95% CI)
Genotypes (N)				
*TT*	95 (74,2)	96 (81,4)	0,17	0,65 (0,35–1,21)
*CT*	30 (23,4)	22 (18,6)	0,35	1,33 (0,72–2,47)
*CC*	3 (2,4)	0	0,09	ND
Alleles (2 N)				
*T*	220 (85,9)	214 (90,7)	0,1	0,62 (0,35–1,1)
*C*	36 (14,1)	22 (9,3)		1,59 (0,9–2,79)

**Table 4:** Genotypes and alleles frequencies of the rs1050504 polymorphism in pulmonary tuberculosis patients and healthy controls

Patients vs Controls				
rs1050504 [C/T]	PTB *N* = 122 (%)	HC N = 141 (%)	*p*-value	OR (95% CI)
Genotypes (N)				
*CC*	66 (54.1)	81 (57.4)	0.58	0.87 (0.53–1.42)
*CT*	51 (41.8)	38 (27)	0.01*	1.95 (1.16–3.27)
*TT*	5 (4.1)	22 (15.6)	0.002*	0.23 (0.08–0.63)
Alleles (2 N)				
*C*	183 (75)	200 (70.9)	0.29	1.23 (0.83–1.81)
*T*	61 (25)	82 (29.1)		

*Significant *p*-values appear in bold

✓ Stratification by sex and age

We also carried out the genetic analysis by sex and age stratification and evaluated the risk for TB development in subjects carrying each relevant genotype. We observed a significant difference in high frequency of the rs1050504 CT genotype in males, but not females, for PTB disease (42.2% vs 25.7%, OR 2.1, 95% CI 1.14–3.88; *p* = 0.01) (Table 5). In contrast, our data show that the TT genotype was more frequent in HC males than in PTB males (4.5% vs 18.8%, OR 0.2, 95% CI 0.06–0.62; *p* = 0.002) (Table 5). Additionally, when taking into consideration the age factor, statistical analysis revealed that the numbers of patients between ages 30 and 49 carrying the rs1050504 TT genotype were significantly fewer than their homologous subjects in the HC group (3.3% vs 6.8%, OR 0.22, 95% CI 0.06–0.82; *p* = 0.002) (Table 6).

**Table 5:** Distribution by sex of allele and genotype frequencies of the rs1050504 and rs2228505 polymorphisms between pulmonary tuberculosis patients and healthy controls

	PTB patients		Controls		TB male vs healthy male		TB female vs healthy female	
	M (%)	F (%)	M (%)	F (%)	*p*-value	OR (95% CI)	*p*-value	OR (95% CI)
rs1050504 [C/T]								
Genotypes	*n* = 90	*n* = 32	*n* = 101	*n* = 40				
*CC*	48 (53,3)	18 (56,3)	56 (55.5)	25 (62.5)	0,76	0,91 (0,51–1,62)	0,59	0,77 (0,29–1,98)
*CT*	38 (42,2)	13 (40,6)	26 (25.7)	12 (30)	0,01*	2,1 (1,14–3,88)	0,34	1,59 (0,6–4,24)
*TT*	4 (4,5)	1 (3,1)	19 (18.8)	3 (7.5)	0,002*	0,2 (0,06–0,62)	0,42	0,39 (0,03–4,02)
Alleles								
*C*	134(74,4)	49 (76,6)	138 (68,3)	62 (77,5)	0,16	1,37(0,87–2,14)	0,89	0,94 (0,43–2,07)
*T*	46 (25,6)	15 (23,4)	64 (31,7)	18 (22,5)				
rs2228505 [T/C]								
Genotypes	*n* = 94	*n* = 34	*n* = 78	*n* = 40				
*TT*	64 (68,1)	31 (91,2)	62 (79,5)	34 (85)	0,09	0,55 (0,27–1,1)	0,41	1,82 (0,41–7,9)
*CT*	27 (28,7)	3 (8,8)	16 (20,5)	6 (15)	0,21	1,56 (0,76–3,17)	0,41	0,54 (0,12–2,38)
*CC*	3 (3,2)	0	0	0	0,11	ND	ND	ND
Alleles								
*T*	155(82,4)	65 (95,6)	140 (89,7)	74 (92,5)	0,53	0,53 (0,28–1,01)	0,43	1,75 (0,42–7,3)
*C*	33 (17,6)	3 (4,4)	16 (10,3)	6 (7,5)				

*ND* not determined; *Significant *p*-values appear in bold

**Table 6:** Distrubition by age of allele and genotype frequencies of the rs1050504 and rs2228505 polymorphisms between pulmonary tuberculosis patients and healthy controls

	≤ 29 years				30–49 years				≥ 50 years			
	PTB (%)	Controls (%)	*p*-Value	OR (95% CI)	PTB (%)	Controls (%)	*p*-Value	OR (95% CI)	PTB (%)	Controls (%)	*p*-Value	OR (95%CI)
rs1050504[C/T]												
Genotypes	*n* = 60	*n* = 44			*n* = 49	*n* = 81			*n* = 13	*n* = 16		
*CC*	31(51,7)	29 (65,9)	0,14	0,55(0,24–1,27)	28 (57,1)	43 (53,1)	0,65	1,17 (0,57–2,4)	8 (61,5)	9 (56,2)	0,91	1,08 (2,23–4,94)
*CT*	27 (45)	12 (27,3)	0,06	2,18 (0,94–5,03)	18 (36,8)	20 (24,7)	0,14	1,77 (0,82–3,82)	5 (38,5)	6 (37,5)	0,82	1,19 (0,25–5,49)
*TT*	2 (3,3)	3 (6,8)	0,41	0,47 (0,07–2,94)	3 (6,1)	18 (22,2)	0,01*	0,22 (0,06–0,82)	0	1 (6,3)	0,37	ND
Alleles												
*C*	89 (74,2)	70 (79,5)	0,36	0,73 (0,38–1,42)	74 (75,5)	106 (65,4)	0,08	1,62 (0,92–2,85)	19 (79,2)	24 (75)	0,71	1,26 (0,35–4,5)
*T*	31 (25,8)	18 (20,5)			24 (24,5)	56 (34,6)			5 (20,8)	8 (25)		
rs2228505[T/C]												
Genotypes	*n* = 64	*n* = 40			*n* = 52	*n* = 62			*n* = 12	*n* = 15		
*TT*	47 (73,5)	31 (77,5)	0,64	0,80 (0,31–2,02)	38 (73,1)	52 (83,9)	0,15	0,52 (0,20–1,30)	10 (83,3)	12 (80)	0,82	1,25 (0,17–9,01)
*CT*	15 (23,4)	9 (22,5)	0,91	1,05 (0,41–2,7)	13 (25)	10 (16,1)	0,23	1,73 (0,68–4,36)	2 (16,7)	3 (20)	0,82	0,8 (0,11–5,77)
*CC*	2 (3,1)	0	0,25	ND	1 (1,9)	0	0,27	ND	0	0	ND	ND
Alleles												
*T*	109 (85,2)	71 (88,7)	0,46	0,72 (0,31–1,69)	89 (85,6)	114 (92)	0,12	0,52 (0,22–1,21)	22 (91,7)	27 (90)	0,83	1,22 (0,18–7,97)
*C*	19 (14,8)	9 (11,3)			15 (14,4)	10 (8)			2 (8,3)	3 (10)		

*Significant *p*-values appear in bold

However, after stratification by sex and age, no evidence of genetic associations between rs2228505 and PTB disease was found.

✓ Haplotype analysis

We did a haplotype analysis for two SNPs: rs1050504 and rs3808607. rs3808607 is located in the promoter region of the *CYP7A1* gene and has been associated with TB in the same population [8].

In healthy individuals, all nine possible diplotype combinations were found. However, only eight diplotypes were found in PTB patients. Interestingly, data analysis found that the CT /AA diplotype was significantly more frequent in PTB patients in comparison to healthy controls and appeared to be associated with an increased risk for the development of pulmonary TB (12% vs. 1%, OR 13.5, 95% CI 1.72–105.9; *p* = 0.0006) (Table 7).

**Table 7:** Distribution of the rs3808607 and rs1050504 diplotype frequencies in pulmonary tuberculosis patients and healthy controls

Diplotypes	PTB Patients (*n* = 68) (%)	Controls (n = 90) (%)	*p*-value	OR (95% CI)
CC/AA	19 (28)	24 (27)	0.43	1.05 (0.56–1.95)
CT/AA	8 (12)	1 (1)	0.0006*	13.5 (1.72–105.9)
TT/AA	0	1 (1)	0.25	Undefined
CC/AC	16 (24)	28 (31)	0.13	0.7 (0.37–1.31)
CT/AC	13 (20)	18 (20)	0.5	1 (0.5–1.99)
TT/AC	1 (1)	1 (1)	0.5	1 (0.06–16.2)
CC/CC	5 (7)	11(12)	0.12	0.55 (0.2–1.46)
CT/CC	5 (7)	5 (6)	0.39	1.17 (0.38–3.64)
TT/CC	1 (1)	1 (1)	0.5	1 (0.06–16.2)

*Significant *p*-values appear in bold

When we analysed the four possible haplotypes, no significant difference was observed between PTB patients and healthy controls (Table 8).

**Table 8:** Distribution of the rs3808607 and rs1050504 haplotype frequencies and LD statistics in pulmonary tuberculosis patients and healthy controls

Haplotype	Haplotype frequencies %		*p*-value	
	PTB	CTRL		
A-C	53	51	0.38	1.08 (0.62–1.89)
A-T	9	4	0.08	2.37 (0.7–7.97)
C-C	25	32	0.13	0.7 (0.38–1.31)
C-T	13	13	0.5	1 (0.43–2.28)
LD statistics	PTB	CTRL		
D’	0.34	0.58		
r^2^	0.05	0.08		

Moreover, the analysis of linkage disequilibrium (LD) revealed that the polymorphisms rs3808607 and rs1050504 were in low LD. The correlation (r2) between these two SNPs in patients and controls was weak (0.05 vs 0.08). In addition, the D’ data also showed evidence of a low LD between these SNPs. In patients, rs3808607 and rs1050504 are coinherited roughly 34% of the time. Therefore, the two SNPs are in weak LD in patients (D’ = 0.34) more than the in control group, where these two polymorphisms are coinherited approximately 58% of the time (D’ = 0.58).

## Discussion

In Morocco, despite efforts that have been deployed, including the setup of a national tuberculosis programme, there is still a long way to go in the fight against TB.

Tuberculosis is a multifactorial disease wherein several parameters in the form of host genetic factors could affect disease outcome. In this regard, numerous studies have reported the crucial role played by the host genetic factors in term of the development of TB disease [27–30].

In the present study, we report for the first time a strong association between the rs1050504 *NSMAF* polymorphism and PTB disease in the Moroccan population. Our data show that the CT genotype is a susceptibility marker for TB and that the mutant TT genotype for rs1050504 plays a protective role against TB. Scarce data has been reported concerning the involvement of this variant on the outcome of TB disease. However, if we take in consideration the particular localization of rs1050504 SNP in 3’UTR (untranslated region) of the *NSMAF* gene, we could generate a hypothesis to explore our finding. In fact, it has been reported in several works that SNPs located in untranslated regions of genes may interfere with mRNA stability and translation, including causing a change in recognition sites for microRNAs (miRNA), RNA-binding proteins, and the polyadenylation machinery [31, 32]. Therefore, 3’UTR variants may result in observed differences in gene expression [32].

In this context, a recent genome-wide association study (GWAS) has reported that the rs1050504-C wild-type allele is located in a putative miRNA binding site for miR-154-3p [33], reported to act as a tumour suppressor in several types of malignancies [34, 35]. In a mutant allele, this miRNA binding site becomes another binding site, the role of which has not yet been explored [33]. This finding is of particular interest, especially when we know that FAN, the protein encoded by *NSMAF*, is implicated in apoptotic signalling [20, 22, 36]. Apoptosis is considered a particular innate defence against Mtb, and it is associated with diminished pathogen viability in infected macrophages [37, 38]. As a result, virulent Mtb strains have developed a capacity to disrupt this mechanism by various means, such as through expression of the anti-apoptotic Bcl-2 (B-cell lymphoma/leukaemia 2) family, which can block the release of cytochrome c from mitochondria [39].

Taken together, we can hypothesize that the presence of the CT genotype could create conditions leading to downregulation of FAN. Conversely, the rs1050504TT SNP could decrease the ability of miRNA to down-regulate FAN, theoretically leading to increased FAN production. In turn, increased FAN could enhance apoptotic signalling to protect against PTB and/or likely affect other roles FAN plays in anti-TB immune response, such as Actin reorganization in macrophages [40] and navigational capacity of leucocytes chemotactic response [41], or its documented role in TNF-α induced neutrophil migration in mouse peritonitis models [21].

However, we cannot exclude the possibility that the association between the rs1050504 SNP and PTB may be secondary to the presence of one or more different variants in close linkage disequilibrium with the *NSMAF* gene or other genes. In particular, this candidate gene is located within the chromosomal region 8q12-q13, characterized by a high LOD score of susceptibility to PTB in the Moroccan population [11]. In addition, more recently, other SNPs located in genes that are closely linked to the *NSMAF* gene, *TOX* (rs2726600 and rs1568952) and *CYP7A1* (rs3808607), have been associated with PTB [8, 42].

After stratification analysis, our data show that the association is maintained only in males but not in females. Males with the rs1050504 CT genotype are more susceptible to PTB, in contrast to those with the TT, which seems to be protective against PTB. These data reinforce the sexual inequality with respect to predisposition to TB; worldwide epidemiological data report that the majority of TB patients are male [43, 44]. This sex discrimination could be due to the sex hormones [43–45]. A previous finding reported that testosterone is immunosuppressive and impairs macrophage activation [46], while oestrogens are pro-inflammatory mediators able to induce the production of TNF-α and stimulate secretion of INF-γ [47, 48].

Interestingly, in our study, the patients between ages 30 and 49 with the TT genotype appeared to be at decreased risk of developing PTB (OR 0.22; *p* = 0.002). Theoretically, the *NSMAF* gene interacts with other genes encoding for cytokines or their receptors that play an essential role in defence against TB. This polygenic aspect of susceptibility to TB might in part explain our finding. When taking into account that genetic variants affecting genes encoding for cytokines or their receptors could be influenced by age and sex, as reported with the AA genotype variant of +874 A/T, affecting the IFN-g gene was associated with active PTB in men (OR 2.42) aged 30–49 years [49].

Analysis of the LD measures revealed that two SNPs (rs3808607 and rs1050504) are in LD. Nevertheless, the values of D’ and r2 indicated that this LD is low. Moreover, our results showed that when the AA rs3806607 genotype and CT rs1050504 genotype are coinherited, the susceptibility to develop TB is strongly significant (OR 13.5; *p* = 0.0006). These data confirm our previous finding wherein the AA rs3808607 genotype of the *CYP7A1* gene is more frequent in PTB patients compared to HC (OR 1.93; *p* = 0.02) [8]. However, the lack of association between rs2228505 and rs36067275 SNPs and PTB in our study, at both the allelic and genotypic levels, could be explained by the absence of the effect of these genetic variants occurring at their positions.

In our study, we report for the first time the allele frequencies of these three SNPs in the Moroccan population, which could be used for others studies in the context of their potential involvement in other diseases. Indeed, data analysis of the rs1050504 SNP revealed that the T mutant allele frequency observed in the Moroccan population (0.29) is close to that reported in Han Chinese (0.32) and Caucasian (0.34) populations and higher than the frequencies observed in Japanese (0.2) and Sub-Saharan African populations (Yoruba) (0.08).

When we analysed the data for the rs2228505-C mutant allele, the allele frequency was 0.09 in Moroccan population, which is similar to that observed in the Sub-Saharan African population (Yoruba) (0.1), but it is much lower than that observed in the Han Chinese and Japanese (0.22, 0.33, respectively) and higher than that in the Caucasian population (CEU) (0.01). Therefore, it will be very interesting to evaluate the impact of the polymorphism of this variant in Chinese and Caucasian TB populations.

Concerning the result of the rs36067275 C > T SNP, we found that the T mutant allele is absent in the Moroccan population as observed in Caucasian (CEU), Han Chinese (HCB) and Japanese (JPT) populations. However, in the Sub-Saharan African population (Yoruba), this allele is present at 0.02 frequency [50].

## Conclusion

In summary, our results suggest that the rs1050504 *NSMAF* SNP may have an impact on the susceptibility or resistance to PTB in the Moroccan population. We are, however, fully aware that the limitation of the current study is its small size. Hence, further studies using larger samples in ethnically diverse populations are needed to better understand the involvement of *NSMAF* in protection against TB. In addition, functional studies are highly recommended in order to evaluate the involvement of the *NSMAF* gene or its product in TB development.

## Abbreviations

3’UTR: Untranslated region
Bcl-2: B-cell lymphoma/leukaemia 2
CI: Confidence interval
HC: Healthy controls
INF-γ: Interferon-gamma
LD: Linkage disequilibrium
Mtb: *Mycobacterium tuberculosis*
NSD: Neutral sphingomyelinase domain
*NSMAF*: Neutral sphingomyelinase activation associated factor
OR: Odds ratio
TB: Tuberculosis
TNFRI: Tumour necrosis factor receptor type I
TNF-α: Tumour necrosis factor-alpha
